# Reduction of chronic non-specific low back pain: A randomised controlled clinical trial on acupuncture and baclofen

**DOI:** 10.1186/1749-8546-5-15

**Published:** 2010-04-24

**Authors:** Jalal Zaringhalam, Homa Manaheji, Ali Rastqar, Maryam Zaringhalam

**Affiliations:** 1 Physiology Department, Neuroscience Research Centre, Shahid Beheshti University of Medical Sciences, Tehran, Iran

## Abstract

**Background:**

Chronic non-specific low back pain (LBP) is a prevalent (80%) and multi-dimensional illness. This study aims to test whether acupuncture, baclofen, or combined treatment with acupuncture and baclofen alleviates symptoms of non-specific chronic LBP in men.

**Methods:**

Eight-four (84) men aged 50-60 years with non-specific chronic LBP were randomly assigned to four groups: the baclofen group received only baclofen (30 mg/day); the acupuncture group received only acupuncture at selected acupoints; the acupuncture + baclofen group received combined treatment with acupuncture and baclofen treatments; and the control group received no pain reduction treatment. After five weeks of treatment, visual analogue scale (VAS) and self-reported pain disability with the Roland-Morris Disability Questionnaire (RDQ) were conducted for outcome measures.

**Results:**

After treatment, the baclofen, acupuncture and acupuncture + baclofen groups all had lower VAS and RDQ scores. Significantly higher reduction and improvement in VAS and RDQ scores were found in the acupuncture and acupuncture + baclofen groups compared to the baclofen group.

**Conclusion:**

The present study indicates that the combined treatment of acupuncture and baclofen is more effective than baclofen treatment alone to reduce pain in patients with non-specific chronic LBP.

**Trial registration number:**

ACTRN12609000698279

## Background

Low back pain (LBP) may be the most prevalent illness, with 80% of the population experiencing it at least once in their lifetime [1]. Up to 90% of all patients with acute LBP recover quickly with or without treatment [2,3]. Ten to forty percent of all LBP cases become chronic which is a burden on the society [4,5]. Furthermore, LBP is a multi-dimensional problem [6] involving pathoanatomical, neurophysiological, physical and psychosocial factors [7]. Most LBP cases are non-specific as definitive diagnosis cannot be established with current radiological methods [4]. The results of research on the effectiveness of treatments for non-specific chronic LBP are inconsistent [8,9]. Some studies suggest that the classification of chronic LBP disorders should be homogenous so that specific interventions tailored for these homogenous groups can be more effective [9]. The most common medications for non-specific LBP are skeletal muscle relaxants and opioid analgesics [8,10]. Muscle relaxants are used to reduce pain of patients with non-specific LBP and, in particular, non-benzodiazepine muscle relaxants such as baclofen are used for symptomatic treatment of chronic LBP [8,11]. Some researchers found that muscle relaxants including baclofen are ineffective [9]. Thus, the use of muscle relaxants for LBP remains controversial. When these drugs are not adequate, another kind of therapy, e.g. acupuncture, is often sought [12,13]. Acupuncture stimulates specific points on the body surface with fine needles [14] and relieves pain in chronic LBP patients as accepted by the World Health Organization (WHO) [15]. Acupuncture treatment may improve the disability of patients with LBP [13,16]. While acupuncture is widely used by patients with chronic LBP, its effectiveness in pain reduction still lacks evidence [12,17,18]. A couple of randomised controlled trials found that combined treatment with acupuncture and baclofen were effective in pain reduction [11,18]; however, the efficacy of this combination has not been demonstrated for non-specific chronic LBP. This study aims to test whether acupuncture, baclofen and combined treatment with acupuncture and baclofen can alleviate symptoms of non-specific chronic LBP in men.

## Methods

### Participants

Men aged 50-60 years with non-specific chronic LBP were recruited through local newspapers except for a few patients (eight people) who had contacted the trial research centres of the Tehran University of Medical Sciences (TUMS). Screening of participants was carried out by a qualified musculoskeletal physiotherapist. Participants met all the following inclusion criteria: (1) lumbar or lumbosacral pain for six months or longer; (2) no radiation of low back pain to other regions; (3) normal neurological signs of lumbosacral nerves including deep tendon and plantar reflexes, voluntary motor function, straight leg raise and sensory function; (4) no acupuncture treatment in the past six months; (5) absence of significant pathology such as bone fracture or severe psychiatric conditions; (6) stable health; and (7) all participants experienced ongoing pain, the intensity of which did not change over the course of a day. Patients were excluded if they had any of the following: (1) major trauma or systemic disorders; (2) conflicting or ongoing co-interventions (drugs and/or alternative treatments); (3) prior use of acupuncture for LBP in the past six months; (4) refusal to be randomised; (5) protrusion or prolapse of one or more intervertebral discs with concurrent neurological symptoms; (6) prior vertebral column surgery; (7) infectious spondylopathy; (8) low back pain secondary to an inflammatory, malignant or autoimmune disease; (9) congenital deformation of the spine (except for slight lordosis or scoliosis); or (10) compression fracture caused by osteoporosis, spinal stenosis, spondylolysis or spondylolisthesis [13,19]. At the first appointment, patient characteristics and baseline measurements were recorded. Part of the screening process relied on self-reported information concerning current medical conditions, medications and serious injuries. Before signing a written informed consent, each participant was given an information sheet explaining the nature of the study. This study was approved by the Ethics Committee of the TUMS.

### Treatments

Based on the information of our planned sample size in previous studies [18] and according to the need for adequate statistical power, a sample size of 84 participants (21 per group) was considered both appropriate and feasible. Participants were randomly assigned to four groups: control (C), acupuncture (AC), baclofen (BA) and baclofen plus acupuncture (BA+AC). Participants were randomised with a stratified blocked randomisation scheme (with random block size of four) and statistical software for randomization (Sample Size version 2.0, Intelligent Masters Company, USA). Block randomization design of 4 (i.e. for every 4 subjects recruited, 1 was assigned in AC group, 1 was assigned in the BA, 1 was assigned in BA+AC group, and 1 was assigned in the C group), was used to ensure balance of the numbers in each group. Stratification was also done based on the initial characteristics shown in Table 1 (VAS and RDQ scores). Participants' assignments were concealed in sealed opaque envelopes that were opened by the acupuncturist before treatment. No assignment was reused with another patient once the envelope had been opened.

**Table 1:** Baseline participant characteristics

Group	Number of participant	Age	Education			Pain duration (years)	BMI	Base RDQ score	Base VAS score
							
			High school or less	Technical school	College graduate				
C	20	54.3 (4.2)	12	2	6	7	31 (3.5)	9.7 (4.4)	64.5 (19.3)
BA	20	55.1 (3.3)	13	2	5	6.7	29.2 (4.2)	9.8 (4.2)	64.5 (18.3)
AC	20	54.2 (5.4)	12	3	5	7.1	32.5 (3.3)	9.6 (3.9)	64.3 (17.8)
BA+AC	20	54.2 (5.6)	13	1	6	6.9	30.3 (4.1)	9.5 (2.8)	64.6 (16.8)
*P*-value		>0.7		>0.05		>0.4	>0.3	>0.5	>0.5

Data are presented as mean (SD).

BMI: body mass index

Treatment course for all groups was five weeks, i.e. standard for chronic pain treatment [19,20]. Control group did not receive any treatment for chronic pain. All participants were advised to maintain their normal lifestyle and not to start any new medications. Acupuncture reporting followed the STandards for Reporting Interventions in Clinical Trial of Acupuncture (STRICTA) [14]. Participants in the AC and BA+AC groups received acupuncture treatment performed by a certified acupuncturist twice a week for five weeks. Acupuncture protocol used in this study was consistent with the neurohumoral mechanism theory of acupuncture [21]. Each patient received needles bilaterally in the following acupoints: *Shenshu *(BL23), *Dachangshu *(BL25), *Panguanshu *(BL28), *Ciliao *(BL32), *Kunlun *(BL60), *Huantiao *(GB30) and *Yanglingquan *(GB34). An aseptic procedure was employed with disposable, stainless 30-gauge needles coupled with electrical stimulation at 4-6 Hz with pulse duration of 0.5 ms [22]. Needles (0.2 mm × 40 mm, Seirin, USA) were inserted into the acupoints until the patient felt dull pain or *deqi*. At each session 10-12 needles were used bilaterally and needles were left in place for 20-25 minutes. Baclofen was orally administered 30 mg/day (15 mg *bid*) which is the recommended effective dose for chronic LBP [23] without causing motor impairment [23]. Patients in the BA+AC group received both baclofen (30 mg/day) and acupuncture for five weeks.

### Outcome measures

Primary outcomes were pain intensity quantified with a 10 cm visual analogue scale (VAS, 0-100 mm) [24] and self-reported pain disability assessed with an Iranian version of the Roland Morris Questionnaire (RDQ, 0-24 points) which is a reliable and valid instrument for measuring functional status in Persian-speaking patients with LBP [25,26]. VAS scores were measured immediately before the first treatment and subsequently at one, two, three, four, five and ten weeks after the first treatment. RDQ scores were measured immediately before the first treatment and subsequently at five and ten weeks after the first treatment. Each VAS or RDQ score was measured immediately before treatment at the specified week [19,27]. The concept of the minimal clinically important difference (MCID) [28], helped interpret changes in VAS and RDQ scores at the individual level. If available, MCID in these outcome measures were defined as a 2-point reduction on VAS and 2.5 points reduction on RDQ.

### Statistical analysis

Data are presented as mean (SD). Statistica software (version 6.0, StatSoft, USA) was used in all statistical analyses. One-way analysis of variance (ANOVA) was performed followed by *post-hoc *Tukey's multiple comparison test (Statistica version 6.0) to determine significant differences in VAS and RDQ scores between groups. Independent t-test was used for comparison of VAS or RDQ scores between two different groups. Statistical significance level was set at *P *< 0.05.

## Results

### Participants

**Figure 1:**
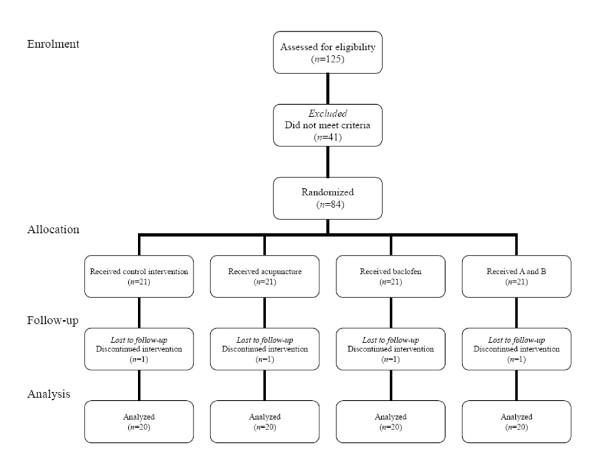
**Participants flow in the study**.

Participants were recruited between May 2006 and February 2008. Of the 125 respondents, 84 (67.2%) met the inclusion criteria for participants. Four participants dropped out from the trial during the treatment due to lack of time (*n *= 2) and pain from acupuncture (*n *= 2). Follow-up measurements and analyses were performed on the remaining 80 participants who completed the study (Figure 1). There was no significant difference in baseline variables such as age, disease, VAS and RDQ scores between groups (Table 1).

### Changes in VAS scores for pain intensity

VAS scores for pain intensity decreased significantly in all treatment groups; however, the exact time course varied (Table 2). The BA group showed a significant decrease in VAS at one and two weeks of treatment compared to baseline (*P *< 0.001) and the control group (*P *= 0.008). Baclofen was more effective in pain reduction in the first week of treatment than in the second week (*P *= 0.04). VAS analysis in the BA group found no significant difference between baselines, three, four, five and ten weeks of treatment. Acupuncture significantly decreased the pain intensity after five weeks of treatment and this effect was stable up to the tenth week of the study. VAS demonstrated a significant decrease at the first week after treatment compared to baseline and control group (*P *= 0.009). VAS in the AC group decreased significantly at two, three, four and five weeks compared to baseline (*P *< 0.001) and one week of treatment (*P *= 0.02). Moreover, acupuncture reduced pain intensity more than baclofen at two, three, four and ten weeks of treatment. The BA+AC group showed a significant reduction in VAS at all time points of this study (*P *< 0.001). VAS scores were significantly lower in the BA+AC group than those in the AC group (*P *= 0.04) (Figure 2).

**Table 2:** Pain intensity scores (VAS) in experimental groups

Group (*n *= 20)	Weeks						
	0	1	2	3	4	5	10
C	64.5 (19.3)	64.2 (21.1)	64.1 (19.4)	64.1 (22.3)	64.3 (20.1)	64.3 (23.8)	64.2 (25.5)
BA	64.5 (18.3)	52.8 (19.4)#	55.1 (21.1)	62.1 (18.7)	63 (20.2)	61.9 (22.3)	63.7 (24.4)
AC	64.3 (17.8)	56.5 (19.9)	50.5 (20.1)#	49.1 (19.3)###	47.3 (18.9)###	47 (19.1)###	50.1 (20.3)###
BA+AC	64.6 (16.8)	52.1 (15.9)‡	45.1 (15.2)‡	45.6 (14.7)‡	42.3 (13.9)‡	40.1 (13.3)‡	47.3 (14.1)‡

Data are presented as mean (SD).

‡ P < 0.05: comparing BA+AC with AC

# P < 0.05, ### P < 0.001: comparing AC with BA

**Figure 2:**
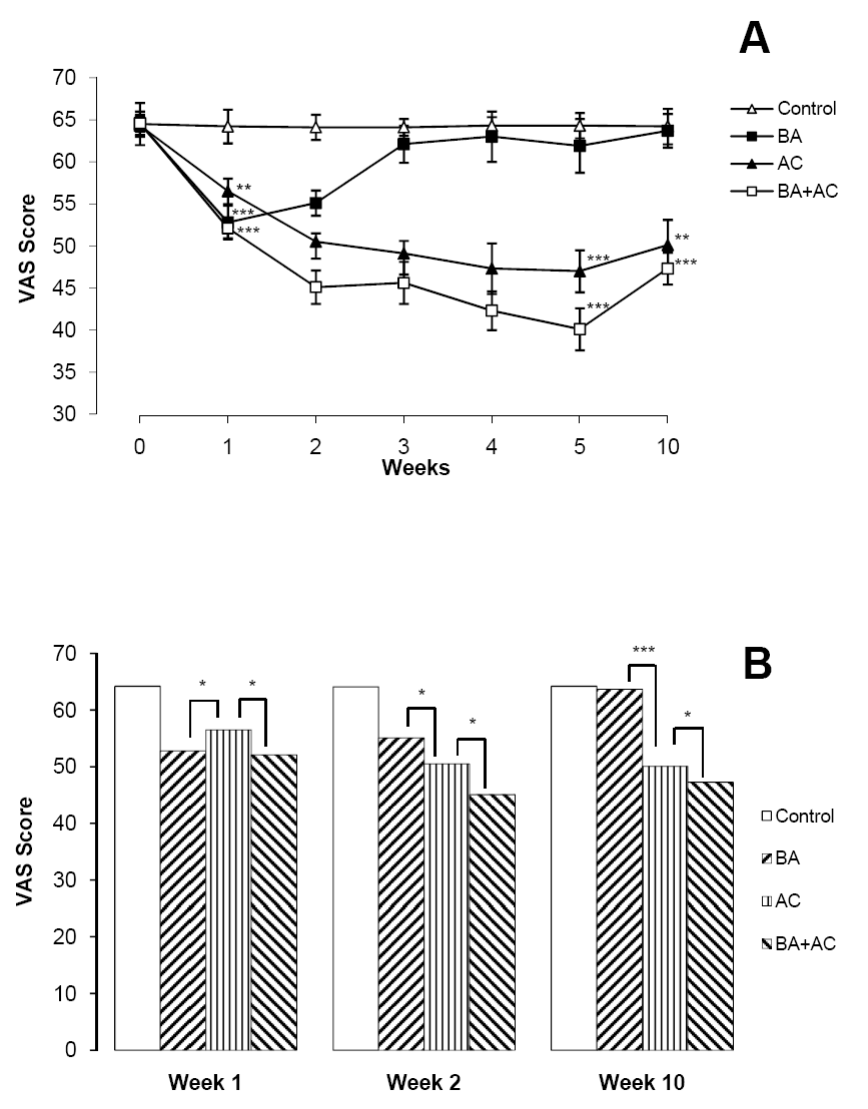
**VAS scores for pain intensity**. (A) Comparison between control and AC, BA or BA+AC. (B) Comparison between AC, BA and BA+AC. * *P *< 0.05. ** *P *< 0.01. *** *P *< 0.001.

### Changes in RDQ scores for pain disability

While the interventions decreased RDQ scores in all groups, the exact time course varied (Table 3). Baclofen administration significantly decreased the RDQ scores after five weeks of treatment compared to zero (*P *= 0.04) and ten weeks (*P *= 0.04). RDQ scores in the AC and BA+AC groups significantly decreased at both five and ten weeks compared to baseline (*P *< 0.001). There were significant decreases in RDQ scores in the AC and BA+AC groups compared to the BA group at five (*P *< 0.001) and ten weeks (*P *< 0.001). RDQ scores in the BA+AC group were also significantly lower than those in the AC group throughout the study (*P *= 0.04) (Figure 3).

**Table 3:** Ronald Morris Questionnaire (RDQ) scores in experimental groups

Group (*n *= 20)	Week		
	0	5	10
C	9.7 (4.4)	9.8 (3.9)	9.9 (4.6)
BA	9.8 (4.2)	8.8 (3.8)	9.5 (4.1)
AC	9.6 (3.9)	6.4 (2.9)‡	7.2 (3.1)‡
BA+AC	9.5 (2.8)	5.7 (1.4)###	5.8###

Data are presented as mean (SD).

‡ *P *< 0.05: comparing AC with BA+AC

### *P *< 0.001: comparing BA+AC with BA

**Figure 3:**
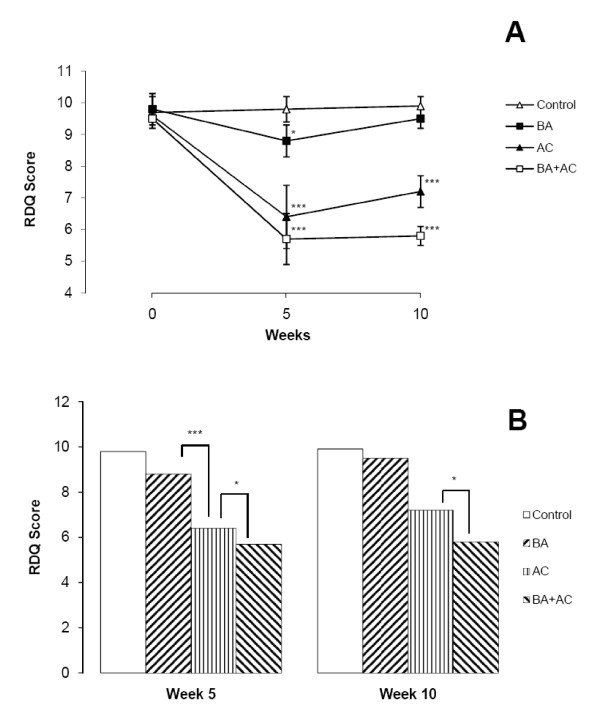
**RDQ scores for pain disability**. (A) Comparison between control and AC, BA or BA+AC. (B) Comparison between AC, BA and BA+AC. * *P *< 0.05. *** *P *< 0.001.

## Discussion

The AC and BA+AC treatments for non-specific chronic LBP were more effective in pain reduction than baclofen treatment alone. Moreover, the anti-nociceptive effects in the AC and BA+AC groups were also more persistent at follow-ups. Acupuncture has demonstrated its potential as a promising treatment for chronic LBP [29,30]. While a number of theories of how acupuncture may treat LBP are available, no accepted mechanism has emerged [30,31]. Similar to descending inhibitory and/or diffuse noxious inhibitory controls in the central nervous system, acupuncture may stimulate the small-diameter afferent fibres, which then reduce the transmission of pain signals thereby inhibiting pain discrimination and perception [32]. Low back muscle spasm and muscle blood flow decrease are the main underlying causes of chronic LBP [33]. Acupuncture alleviates tension and improves blood flow in the treated muscles [34]. Thus, acupuncture treatment may improve lumbar function and reduce pain via increasing the blood flow to the affected region [35]. Non-benzodiazepine muscle relaxants are often used to treat non-specific LBP [36], as a gamma-aminobutyric acid (GABA) derivative with central nervous system action and a substance P antagonist [37,38]. In this study baclofen (30 mg/day *per oral*) reduced the pain intensity but only effective in the first two weeks. These results are in line with a previous study that did not find significant and consistent decrease in pain intensity with baclofen treatment for chronic spastic pain [39]. Due to the controversies some practitioners are reluctant to prescribe baclofen to their patients [9]. Baclofen is effective for immediate pain relief [24], whereas acupuncture is effective to treat long-term pain and alleviate pain-related disabilities [27,40,41]. As baclofen treatment alone does not produce major functional benefits [42], combination treatment maybe an alternative [43]. This RCT does show that the BA+AC group had lower VAS and RDQ scores than other groups, i.e., the combined acupuncture and baclofen treatment is more effective to treat non- specific chronic LBP than either treatment alone [44].

## Conclusion

The present study indicates that the combined treatment of acupuncture and baclofen is more effective than baclofen treatment alone to reduce pain in patients with non-specific chronic LBP.

## Abbreviations

LBP: Low back pain; VAS: Visual analogue scale; RDQ: Roland-Morris Disability Questionnaire; NSAIDs: Non-steroidal anti-inflammatory drugs; WHO: World Health Organization; TUMS: Tehran University of Medical Sciences; C: Control; AC: Acupuncture; BA: Baclofen; BA+AC: Baclofen plus acupuncture; STRICTA: STandards for Reporting Interventions in Clinical Trial of Acupuncture; BL: Bladder; GB: Gallbladder; SD: Standard deviation; MCID: Minimal clinically important difference; ANOVA: One-way analysis of variance; GABA: Gamma-aminobutyric acid; RCT: Randomised controlled trial.

## Competing interests

The authors declare that they have no competing interests.

## Authors' contributions

JZ designed the study, performed the acupuncture treatment and drafted the manuscript. HM conceived the study and participated in the sequence alignment of the study. AR designed the study and performed the statistical analysis. MZ coordinated the study. All authors read and approved the final version of the manuscript.
